# Continuous Data-Driven Monitoring in Critical Congenital Heart Disease: Clinical Deterioration Model Development

**DOI:** 10.2196/45190

**Published:** 2023-05-16

**Authors:** Ruben S Zoodsma, Rian Bosch, Thomas Alderliesten, Casper W Bollen, Teus H Kappen, Erik Koomen, Arno Siebes, Joppe Nijman

**Affiliations:** 1 Department of Paediatric Intensive Care University Medical Center Utrecht Utrecht Netherlands; 2 Department of Anaesthesiology University Medical Center Utrecht Utrecht Netherlands; 3 Department of Information and Computing Sciences Utrecht University Utrecht Netherlands

**Keywords:** artificial intelligence, aberration detection, clinical deterioration, classification model, paediatric intensive care, pediatric intensive care, congenital heart disease, cardiac monitoring, machine learning, peri-operative, perioperative, surgery

## Abstract

**Background:**

Critical congenital heart disease (cCHD)—requiring cardiac intervention in the first year of life for survival—occurs globally in 2-3 of every 1000 live births. In the critical perioperative period, intensive multimodal monitoring at a pediatric intensive care unit (PICU) is warranted, as their organs—especially the brain—may be severely injured due to hemodynamic and respiratory events. These 24/7 clinical data streams yield large quantities of high-frequency data, which are challenging in terms of interpretation due to the varying and dynamic physiology innate to cCHD. Through advanced data science algorithms, these dynamic data can be condensed into comprehensible information, reducing the cognitive load on the medical team and providing data-driven monitoring support through automated detection of clinical deterioration, which may facilitate timely intervention.

**Objective:**

This study aimed to develop a clinical deterioration detection algorithm for PICU patients with cCHD.

**Methods:**

Retrospectively, synchronous per-second data of cerebral regional oxygen saturation (rSO_2_) and 4 vital parameters (respiratory rate, heart rate, oxygen saturation, and invasive mean blood pressure) in neonates with cCHD admitted to the University Medical Center Utrecht, the Netherlands, between 2002 and 2018 were extracted. Patients were stratified based on mean oxygen saturation during admission to account for physiological differences between acyanotic and cyanotic cCHD. Each subset was used to train our algorithm in classifying data as either stable, unstable, or sensor dysfunction. The algorithm was designed to detect combinations of parameters abnormal to the stratified subpopulation and significant deviations from the patient’s unique baseline, which were further analyzed to distinguish clinical improvement from deterioration. Novel data were used for testing, visualized in detail, and internally validated by pediatric intensivists.

**Results:**

A retrospective query yielded 4600 hours and 209 hours of per-second data in 78 and 10 neonates for, respectively, training and testing purposes. During testing, stable episodes occurred 153 times, of which 134 (88%) were correctly detected. Unstable episodes were correctly noted in 46 of 57 (81%) observed episodes. Twelve expert-confirmed unstable episodes were missed in testing. Time-percentual accuracy was 93% and 77% for, respectively, stable and unstable episodes. A total of 138 sensorial dysfunctions were detected, of which 130 (94%) were correct.

**Conclusions:**

In this proof-of-concept study, a clinical deterioration detection algorithm was developed and retrospectively evaluated to classify clinical stability and instability, achieving reasonable performance considering the heterogeneous population of neonates with cCHD. Combined analysis of baseline (ie, patient-specific) deviations and simultaneous parameter-shifting (ie, population-specific) proofs would be promising with respect to enhancing applicability to heterogeneous critically ill pediatric populations. After prospective validation, the current—and comparable—models may, in the future, be used in the automated detection of clinical deterioration and eventually provide data-driven monitoring support to the medical team, allowing for timely intervention.

## Introduction

Critical congenital heart disease (cCHD)—requiring a cardiac intervention (cardiac surgery or therapeutic cardiac catheterization) in the first year of life for survival—globally occurs in 2-3 of every 1000 live births [1-3]. In the critical perioperative period, intensive multimodal monitoring at a pediatric intensive care unit (PICU) is warranted as their organs, especially the brain, may be severely injured due to changes in blood flow and oxygenation caused by hemodynamic and respiratory events [4-7]. As such, clinical data streams that include regional cerebral oxygen saturation (rSO_2_) using near-infrared spectroscopy, as well as vital parameters (eg, heart rate and blood pressure), are continuously acquired in these critical patients and produce substantial amounts of high-frequency data for medical assessment purposes.

However, integrated assessment of these clinical data streams—condensing data to comprehensible information—can be especially challenging in the cCHD population due to their unique and dynamic physiology. For example, an oxygen saturation (SpO_2_) varying from 60% to 90% can be normal in some forms of cyanotic cCHD, such as hypoplastic left heart syndrome [8], where it can be deadly in different forms of cCHD. Adding up to the challenge, the overall intensive care unit and PICU architecture is increasingly shifting toward single-person rooms, promoting privacy and family-centered care. However, this also results in decreased immediate visibility of the patient and subsequently raises the threshold to combine monitoring data with hands-on bedside input (ie, visual, tactile, and response to stimuli).

With the rapid growth in both computing power and data storage over the last decade, the potential benefits of advanced data science algorithms, such as machine learning (ML), have greatly increased for health care [7,9-11]. Clinicians may benefit from the ML-assisted continuous interpretation of these large quantities of monitoring data at the PICU, as it can provide them with data-driven remote monitoring support through automated detection of clinical deterioration. At times of suspected deterioration, staff may be notified in a timely manner, allowing for medical evaluation and possible treatment in an effort to reduce the risk of injury.

Most of the previously published models aimed at providing data-driven monitoring support do so through a prognostic early warning score for a certain population and consider both static (eg, diagnosis or age) and dynamic (eg, vital signs) parameters. These were recently reviewed by Muralitharan et al [10] and included postoperative patients or those in step-down wards [12,13], emergency departments [14,15], and adult intensive care [16,17].

To date, ML-based early warning algorithms in the pediatric population are overall scarce (eg, Park et al [18]) and very sporadic in the congenital heart disease (CHD) population in the PICU (eg, Ruiz et al [19]), whereas none have been reported as being currently in use. In the specific case of cCHD, the heterogeneity of the population, both with respect to the normal values in different age groups [20] and the spectrum of underlying diseases, together with the limited amount of critically ill pediatric patients, provide substantial challenges for the application of advanced data science [21].

This study aimed to develop a diagnostic model using transparent ML, which is capable of continuously detecting clinical deterioration in patients with cCHD admitted to the PICU while considering their unique hemodynamic physiology. The model’s internal architecture is demonstrated, its performance evaluated in comparison to expert opinion, and the future implementation discussed, along with recommendations provided for similar research.

## Methods

### Patient Population and Parameters

Infants younger than 1 year with cCHD admitted perioperatively to the PICU of the University Medical Centre Utrecht between 2002 and 2018 were included based on the availability of time-synchronous data streams. We collected data from 5 vital parameters in a frequency of 1 measurement per second, namely SpO_2_, regional cerebral saturation (rSO_2_) in both hemispheres, invasive mean arterial blood pressure (IBP), respiratory rate (RR), and heart rate (HR), as well as current mechanical ventilation status. Patients were excluded if less than 12 hours of complete data were available or due to low birth weight (<2000 g). 

### Ethical, Distributional, and Guideline Statements

As fully anonymized data were used, the medical ethical review committee of the Wilhelmina’s Children Hospital waived informed consent (application number 22/822). In manuscript preparation, the Transparent Reporting of a Multivariable Prediction Model for Individual Prognosis or Diagnosis (TRIPOD) checklist [22] was used (Multimedia Appendix 1).

### Data Preprocessing

RR was measured through thoracic movement as a result of electrocardiographic impedance derivation with the electrocardiographic leads from the Philips Intellivue MP70 monitor. Because infants, especially neonates, can have considerable fluctuations of RR within minutes, a trend movement was examined rather than absolute values: a 300-second moving average for RR was implemented preceding each time point *t.* Cerebral rSO_2_ was measured with near-infrared spectroscopy with the Medtronic INVOS 5100 monitor using 2 pediatric cerebral sensors. If both probes recorded a value, their mean was used in model calculations. At our institution, end-tidal carbon dioxide (EtCO_2_) is considered in all mechanically ventilated patients to monitor the efficacy of ventilation; therefore, EtCO_2_ was extracted to determine the current mechanical ventilation status at each time point (ie, currently mechanically ventilated if EtCO_2_>0 at time *t*). No imputation was performed to account for missing values in these parameters. To account for the underlying varying physiology of CHD, patients were stratified into 2 subsets based on average SpO_2_ during admission (ie, <90% versus ≥90%), as measured with oximetry using the Philips Intellivue MP70 monitor (FAST technology with the Nelcor sensor). As SpO_2_ is a parameter in the model and therefore directly influences predictive performance, we decided to use data-driven stratification of CHD in order to accurately represent the spectrum of underlying diseases throughout the stratified group regardless of clinical diagnosis.

### Model Architecture

**Figure 1:**
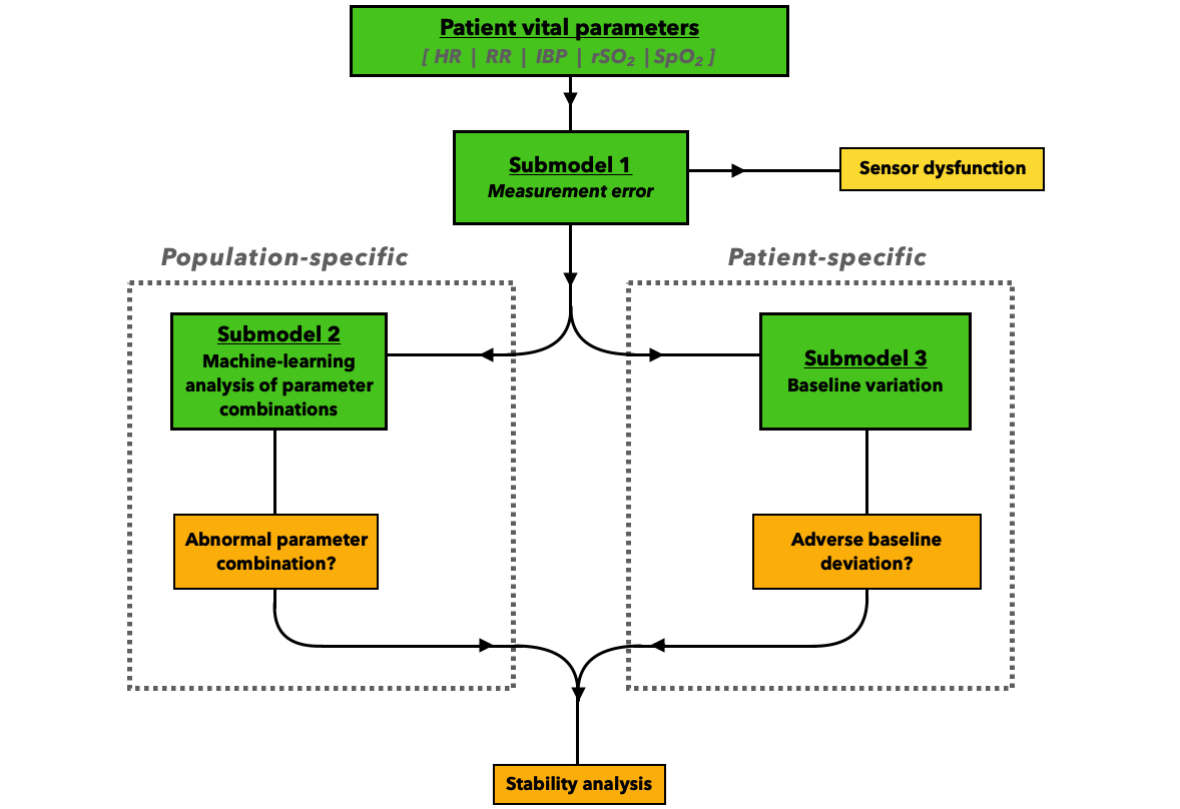
Flowchart depicting the model’s analytic process of detecting deterioration through submodel 1 (sensor dysfunction), submodel 2 (machine learning analysis of parameter combinations), and submodel 3 (analysis of baseline deviations). HR: heart rate; IBP: invasive mean blood pressure; RR: respiratory rate; rSO_2_: regional cerebral oxygen saturation; SpO_2_: oxygen saturation.

To facilitate future clinical use, our model was developed using explainable methods (ie, through methods allowing clinicians to understand what features and assets contribute to the output) as opposed to the so-called “black box” models (eg, deep neural networks), where their methodological foundation and feature derivation remains beyond grasp to most clinicians. The model’s internal architecture consisted of 3 separate models, which were integrated to coordinate a classification response of either sensor dysfunction or stable or unstable patient status (Figure 1). Each of the 3 models relied on a specific analysis: sensorial dysfunction (submodel 1 in Figure 1), classification of normal and abnormal vital parameter combinations (submodel 2 in Figure 1), and detection and analysis of significant patient-specific baseline deviations (submodel 3 in Figure 1).

An analyzed time point *t* was deemed unstable if no sensorial dysfunction was detected, and either submodel 2 or 3—or both—classified the time point *t* to be unstable. The continuous data points were converted to episodes through a 5-minute moving time frame, where an episode was considered unstable when classified thus in at least 4 minutes (ie, ≥80%) out of any 5-minute time frame. If less than 4 (nonconsecutive) minutes of the episode (ie, <80%) were deemed unstable, the time frame was consequently classified as stable. To allow for baseline build-up (submodel 3), the first hour of admission was analyzed without triggering a classification response.

All models were built using RStudio (version 1.4; R Foundation for Statistical Computing). The packages used in construction, as well as the source code, can be found on the website of our research group [23]. 

### Submodel 1: Sensor Dysfunction

To reduce the faulty classification of patient status due to sensor errors, dysfunctions of IBP, SpO_2_, rSO_2_, and RR were evaluated. HR sensor dysfunction was not included as no reliable distinction between, for example, cardiac arrest (HR=0 beats per minute) and sensor error, could be made. IBP and SpO_2_ dysfunction was determined as a difference of >25 points on their respective scales compared to the previously measured value (at time point *t*–1)*.* The lower and upper limits of the rSO_2_ scale (≤15% and ≥95%, respectively) were noted as measurement error as these values are unlikely to be a valid measurement and rather emerge due to escaping sensor-light emission. An RR sensor malfunction was considered to be a rate below 5 breaths per minute. Upon detection, measurements in the minute preceding the first detection (at time point *t*–60 seconds) up to the minute proceeding (at time point *t*+60 seconds) the last detection (*t*) were considered unfit for adequate classification and consequently classified as sensor dysfunction. 

### Submodel 2: Machine Learning Analysis of Parameter Combinations

**Figure 2:**
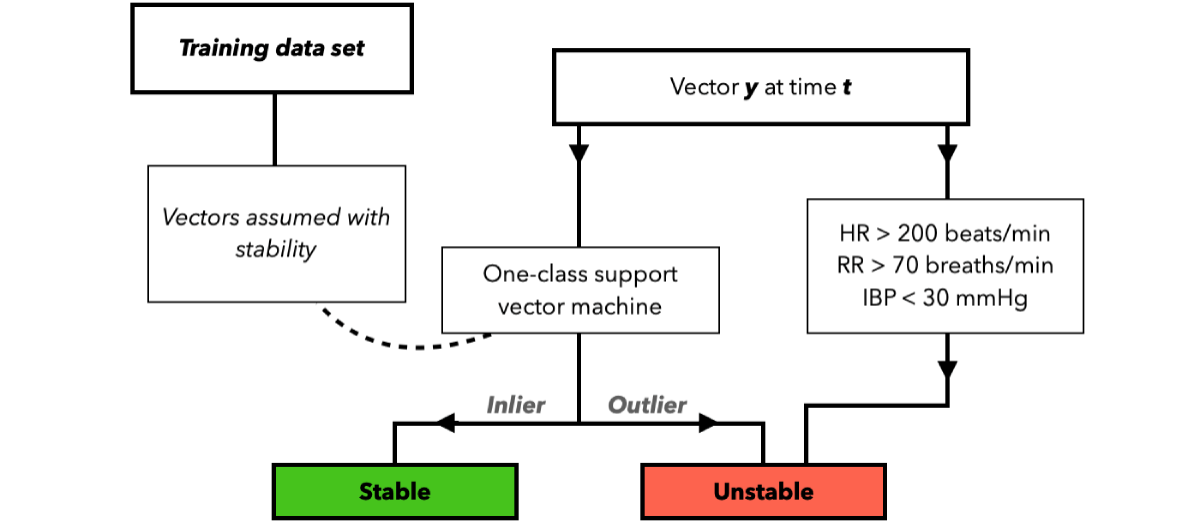
Flowchart depicting the layout of submodel 2, where stability and instability is detected through both support vector machine learning of population-specific parameter instability as well as through predefined static cutoff values of HR, RR, and IBP. HR: heart rate; IBP: invasive mean arterial blood pressure; RR: respiratory rate.

Combinations of parameters were analyzed and classified to either be stable or unstable. Each vector of the parameters (RR, HR, IBP, rSO_2_, and SpO_2_) was normalized and reduced to a single principal component using the Mahalanobis method [24], with respect to the stratified subset–specific (SpO_2_<90% versus SpO_2_≥90%) mean, variance, and correlation matrices (Multimedia Appendix 2). Vectors with a corresponding Mahalanobis distance greater than the 80th percentile were deemed unstable and discarded from the subset. The remaining vectors were divided into a random 80:20 train:test partition and used to train a one-class support vector machine (SVM). We used a square-exponential radial basis function kernel with a 5% soft margin (µ) to prevent overfitting. As the SVM was trained using, presumably, stable vectors of parameters, any nonresemblant vector was classified as unstable by the SVM. Additionally, singular parameters were considered unstable when exceeding static cutoff values determined by the consensus of pediatric intensivists (Figure 2).

### Submodel 3: Baseline Variation

**Figure 3:**
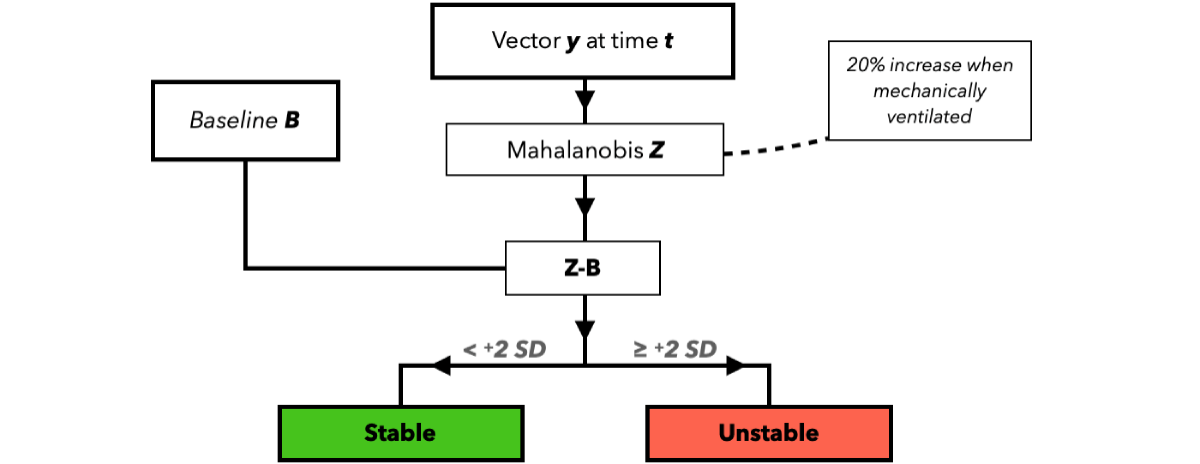
Flowchart depicting the layout of submodel 3 in the process of determining stability through baseline deviation analysis.

Baseline variation analysis focused on the detection of abnormal parameter deviations in comparison to the patient’s unique baseline (Figure 3). A vector of parameters (HR, RR, IBP, rSO_2_, and SpO_2_) was reduced to a single principal component using the earlier introduced Mahalanobis distance [24] and increased by 20% at times of mechanical ventilation (ie, EtCO_2_>0) due to the consequent iatrogenically diminished variation in parameters. The current Mahalanobis trend (Z)*,* through a 300-second moving median preceding time point *t*, was subsequently compared to the patient’s unique baseline (B, median of all Mahalanobis distances preceding *t*). As Mahalanobis distance is calculated using normalized values, trend movement toward subset mean values (Z–B<0) was assumed to be related to clinical improvement, where a significant trend drifting ≥2 SDs from both the subset mean values, as well as the baseline, was deemed to result from instability (Z–B≥+2SD). SD was calculated after the removal of the upper 20th percentile of the baseline corrected Mahalanobis distance (ie, the SD in supposedly stable time points) with respect to chronologicity*.*

### Model Performance

Novel, unseen data from the 5 parameters (HR, RR, IBP, SpO_2_, and rSO_2_), along with the model’s classification, were visualized in detail (Multimedia Appendix 3). Two experienced pediatric intensivists (JN and EK) reviewed these charts, each noting independently, being blinded from each other, whether they agreed with the model classification. Any difference in opinion was resolved by an independent third expert. The performance of the algorithm was consequently based on expert opinion, noting both time-percentual correctness as well as episodic performance. Episodes were counted with a maximum duration of 2 consecutive hours to prevent shifting results based on episode length. 

## Results

### Patient and Parameter Characteristics

In total, 92 patients were initially identified with time-synchronized parameters in their data sets, of whom 14 (15%) were excluded (<12-hour data: n=11, 79%; birthweight<2000 g: n=3, 21%). The remaining 78 patients were stratified into 2 subgroups based on mean SpO_2_ during admission: SpO_2_<90% (n=26, 33%) and SpO_2_≥90% (n=52, 67%). The group characteristics are shown in Table 1. A list of cardiac diagnoses and performed surgical interventions on included patients is provided in Multimedia Appendix 4.

**Table 1:** Baseline characteristics of stratified subsets with an average oxygen saturation (SpO_2_) of <90% versus those with an SpO_2_ of ≥90%.

Characteristics			SpO_2_<90% (n=26)		SpO_2_*≥*90% (n=52)
**Study population**					
	Male gender, n (%)	21 (81)		35 (67)	
	Birth weight (kg), median (IQR)	3.4 (3.1-4.0)		3.3 (3.0-3.6)	
	Age at *t*=0 (days), median (IQR)	7.0 (2.3-11)		9.0 (5.0-17.3)	
	Available data (hours), median (IQR)	63.1 (46.4-98.9)		44.0 (23.8-61.6)	
**Vital parameters, median (IQR)**					
	Heart rate (beats per minute)	159 (147-170)		146 (132-158)	
	Respiratory rate in (breaths per minute)	34 (30-38)		35 (30-40)	
	SpO_2_ (%)	77 (70-81)		97 (95-100)	
	Regional cerebral oxygen saturation (%)	55.0 (49.0-63.0)		71.5 (63.5-80.0)	
	Mean Invasive blood pressure (mm Hg)	51 (47-57)		53 (47-60)	

### Model Performance

A total of 209 hours of data from 10 patients across the SpO_2_<90% group (n=5, *t*=98 hours) and SpO_2_≥90% group (n=5, *t*=111 hours) were classified by our algorithm for performance analysis.

#### Patients With an Average SpO_2_ of <90%

In the subgroup with an average SpO_2_ of <90%, a total of 77 stable episodes occurred, where 66 (86%) were correctly classified. These 77 episodes lasted 90 hours, where 87 (97%) hours were correctly analyzed. Unstable episodes occurred 21 times for a total of 8 hours. In total, 17 (81%) of these episodes were correctly classified, adding up to 4 (51%) hours. Further, 2 (12%) of the unstable episodes were correctly detected; yet, algorithmic labeling did not cover the full length of the episode.

#### Patients With an Average SpO_2_ of ≥90%

Across the subgroup with an average SpO_2_ of ≥90%, stable episodes occurred 76 times, of which 68 (89%) were correctly classified. Stable episodes lasted a total of 91 hours, where 84 (92%) hours were correctly classified. Across 36 unstable episodes adding up to 20 hours, 18 (86%) hours in 29 (81%) episodes were classified accordingly. Out of the 29 correctly detected unstable episodes, 8 (28%) were partially correct.

#### Overall Performance

Considering both groups, 134 of the 153 (88%) stable episodes were correctly labeled (171 of 181 hours, 93%). Unstable episodes were correctly labeled in 46 of the 57 (81%) observed episodes (22 of 29 hours, 77%). A total of 12 unstable episodes were missed by the model in testing. Sensor dysfunction occurred a total of 138 times, of which 130 (94%) were accurately labeled (Table 2).

**Table 2:** Performance analysis overview of the aberration detection algorithm when compared to expert consensus, depicted in either episodic or time occurrence.

Model performance			SpO_2_^a^<90% (n=5)		SpO_2_*≥*90% (n=5)		Total (n=10)	
**Stable moment**								
	Episodic occurrence, n	77		76		153		
	Episodic correctness (%), n (%)	66 (86)		68 (89)		134 (88)		
	Time occurrence (hours), n	90		90		181		
	Time correctness (hours), n (%)	83 (92)		84 (93)		171 (93)		
**Unstable moment**								
	Episodic occurrence, n	21		36		57		
	Episodic correctness, n (%)	17 (81)		29 (81)		46 (81)		
	Time occurrence (hours), n	8		20		29		
	Time correctness (hours), n (%)	5 (63)		17 (83)		22 (77)		
**Sensor dysfunction**								
	Episodic occurrence, n	57		81		138		
	Episodic correctness, n (%)	56 (98)		74 (91)		130 (94)		

^a^SpO_2_: oxygen saturation.

## Discussion

### Principal Findings

In this proof-of-concept study, we have developed and retrospectively evaluated an advanced data science algorithm for PICU patients with cCHD aimed at automated detection of clinical deterioration during their critical perioperative period. Through 2-fold analysis of vital parameters, both in relation to each other and in comparison to the patient’s unique baseline parameters, a tailored approach was demonstrated to monitor complex and hemodynamically challenging patients. Overall, our model accurately detected clinical stability and deterioration in, respectively, 88% and 81% of expert-confirmed episodes. Sensor dysfunction occurred 138 times, of which 94% were rightfully detected.

### Clinical Relevance

The population of patients with cCHD has been shown to be at substantial risk of deterioration in their perioperative period, as they are susceptible to a range of hemodynamic and respiratory events, especially in the postoperative period [4-7]. These disturbances in (cerebral) blood flow and oxygenation may eventually result in damage to internal organs, such as the gut and the brain [7,25]. Brain injury, for example, is observed in up to 60% of postoperative patients with cCHD and is known to cause severe neurodevelopmental impairment, significantly impacting quality of life [26,27]. Adequate detection of patient deterioration could facilitate timely intervention and may, eventually, prevent the onset of novel (brain) injury. However, adequate and timely detection of ongoing deterioration is becoming increasingly difficult through the ever-growing amount of complex and dynamically interpretable data inherent to the cCHD population, posing a 24/7 monitoring challenge to the medical team. Additionally, previous research has noted subtle variations in vital parameters to precede adverse events [7] as well as significant phenotype differences in cCHD related to an adverse outcome [4]. Through mixed-effects regression analysis, Nicoll et al [4] described independent associations between elevated HR (*P*=.003) and elevated systolic BP (*P*=.02) with novel brain injury in the first 72 hours after surgery. These physiological differences were most significant directly postoperatively and decreased with time, again highlighting the importance of adequate and intensive perioperative monitoring to identify patients at higher risk of deterioration. However, paying attention to these different physiological phenotypes and subtle parameter variations requires 24/7 vigilance from staff, greatly increasing their cognitive load. With algorithmic condensation of clinical data streams toward comprehensible information, the cognitive load on clinicians and nurses will likely be decreased, providing support to both patients and the medical team.

### Comparison to Previous Work

Overall, research classifying current patient status in CHD—rather than predicting a future adverse event—is very scarce. To the best of our knowledge, diagnostic AI models classifying current patient status in CHD and cCHD have yet to be published. A fair comparison of predictive versus diagnostic models in CHD is limited due to their different aims and setup; however, their methodological comparison is possible to some degree.

In 2013, Clifton et al [14] proposed an algorithm for adults in the emergency department through the use of an integrated monitoring system that combines high-frequency physiological data to predict upcoming escalation of care. Here, they have developed and tested several ML methods against an existing evidence-based early warning score. The different approaches to predicting escalation of care had mixed results, where the SVM had a high detection rate (>85%, time frame–dependent), yet, also, a high false positive rate (27%). If their algorithm were applied to, for example, the population of patients with cCHD, their inherent dynamic circulation would not be taken into account, most likely decreasing the detection rate.

In this study, it is argued that the 2-fold analysis of stability (ie, parameters in relation to each other and with different time points) is of significant value to the monitoring or predicting of outcomes in heterogeneous populations, such as pediatrics, using high-frequency physiological data. As such, future studies aiming to monitor, classify, or predict outcomes in the pediatric population are encouraged to evaluate the need for adjustment to their patients’ dynamic physiology and consider their model’s resilience to these dynamic conditions. However, it must also be acknowledged that robust statistical methods for transparent advanced data science models, such as those proposed in this study, remain scarce to this date, especially in complex clinical time-series data. 

Additionally, a multitude of “black box models” (eg, deep neural networks) have shown spectacular results in various fields, including the prediction of clinical deterioration [10,16,19]. In 2022, Ruiz et al [19] demonstrated their retrospective data-driven extreme gradient boosted model aimed at predicting clinical deterioration (defined as adverse events, such as intubation, cardiopulmonary resuscitation or initiation of extracorporeal membrane oxygenation) in cCHD over a time frame up to 8 hours. Through the model’s assessment of 1028 variables (eg, medication, vital parameters, laboratory values, etc), they have achieved accurate predictions and good calibrations with at least 4 hours prior to intubation (area under the receiver operating characteristic curve 0.927, 95% CI 0.825-0.994) or cardiopulmonary resuscitation and extracorporeal membrane oxygenation (area under the receiver operating characteristic curve 0.914, 95% CI 0.796-0.991).

However, the methodological foundations of such complex models remain beyond the grasp of most clinicians. It is likely that models with explainable methods are more likely to be implemented in daily practice and, therefore, explainable modeling techniques were used in this study. The clinical usefulness of our proof of concept, however, has yet to be proven as it is currently limited by its underpowered sample size and the retrospective analysis of model performance. In the near future, the model will be trained and evaluated on a more heterogeneous population to increase performance and versatility, boosting the chances of successful (external) validation while maintaining a sharp clinical perspective: how can the algorithm be most valuable to both patients (eg, early intervention and reduced risk of injury) as well as the medical team (eg, reduced cognitive load)? 

### Strengths and Limitations

Several other limitations to this proof-of-concept study must be addressed. First (and foremost), selection bias was introduced through the inclusion of patients with cerebral rSO_2_ measurements, as well as IBP. Cerebral rSO_2_ monitoring is currently not available as a standard of care in global (cardiac) PICUs, and as such, the clinical value of our model will decrease outside the research institution. Additionally, a relatively high sample rate of 1 Hz was used to extract data. As not all parameters are transmitted at the same frequency, internal sampling or resampling is inevitable, possibly affecting data quality.

Second, we have chosen a retrospective approach to analyze model performance. Analyzing patient stability solely based on retrospective parameters remains particularly challenging, even for medical experts. Increases or decreases in parameter values may, for example, originate for a number of reasons, such as feeding or movement, and may have little to no clinical significance. The classification of episode stability or sensor dysfunction was evaluated by expert consensus based on the same data available to the model. However, no hard judgments can be made on the clinical relevance of that episode, as the data were not labeled prospectively (ie, containing labeled events). Arguably, prospective validation with members of the medical team performing a simultaneous bedside evaluation on agreement with the model will be one of the future goals.

Third, in the stability analysis of parameter combinations, an SVM was trained to recognize stability across 5 dimensions. In selecting presumably stable parameter combinations, an 80th percentile split of the vector’s corresponding Mahalanobis distance was made, partially based on earlier work by Clifton et al [14]. However, since no explicit labeling was possible in the data set, the chosen cutoff percentile remains arbitrary. Additionally, through the use of normalized data, an assumption is made that any deviation from subset-specific mean values reflects an adverse development. However, for some parameters, an increase or decrease does not necessarily reflect an adverse event, which may result in an overestimation of clinical status and aid in the induction of alarm fatigue. Future research may point out different methods to be more effective.

### Future Directions

Several steps must be taken to progress this model—and others alike—toward implementation in daily clinical practice [28,29]. Primarily, a data infrastructure is required to enable real-time or near–real-time data availability to AI models, allowing their prospective validation. In the near future, such a platform will be constructed, speeding up the qualitative performance analysis of data science models while promoting guideline adherence, such as the TRIPOD guidelines [22]. Eventually, AI models will be implemented into the daily workflow, aiding the medical team and likely decreasing their cognitive load, which is beneficial for, in this instance, the continuous interpretation of clinical data streams in hemodynamically challenging patients.

### Conclusions

In this study, a proof-of-concept algorithm aimed at detecting clinical deterioration in patients with cCHD at the PICU was developed and retrospectively evaluated, achieving reasonable performance considering the heterogeneous population of neonates with cCHD. Combined analysis of baseline (ie, patient-specific) deviations and simultaneous parameter-shifting (ie, population-specific) proofs to be promising with respect to enhancing applicability to heterogeneous critically ill pediatric populations.

Although performance should be improved and prospectively validated, advanced data science models such as the one presented here may, in the future, be used in automated detection of clinical deterioration, providing real-time data-driven monitoring support in the case of hemodynamically challenging patients and allowing for timely intervention.

## Appendix

Multimedia Appendix 1 TRIPOD (Transparent Reporting of a Multivariable Prediction Model for Individual Prognosis or Diagnosis) checklist.

Multimedia Appendix 2 Mean, standard deviation, and correlation matrices of stratified subgroups.

Multimedia Appendix 3 Clinical deterioration detection visualized.

Multimedia Appendix 4 Overview of cardiac diagnosis and performed surgical procedures of included patients.

## Abbreviations

cCHD: critical congenital heart disease
CHD: congenital heart disease
EtCO_2_: end-tidal carbon dioxide
HR: heart rate
IBP: invasive mean arterial blood pressure
ML: machine learning
PICU: pediatric intensive care unit
RR: respiratory rate
rSO_2_: regional cerebral oxygen saturation
SpO_2_: oxygen saturation
SVM: support vector machine
TRIPOD: Transparent Reporting of a Multivariable Prediction Model for Individual Prognosis or Diagnosis

## Data Availability

The data sets presented in this paper are not readily available due to confidentiality restrictions preventing their distribution. Requests to access the data may be directed to the corresponding author.
