# Supplementary material for: Continuous Data-Driven Monitoring in Critical Congenital Heart Disease: Clinical Deterioration Model Development
Source: JMIR Cardio. 2023 May 16;7:e45190. doi: 10.2196/45190 (PMC10230358; doi:10.2196/45190)
Supplement: Multimedia Appendix 2 [file cardio_v7i1e45190_app2.docx]

***Appendix 2, Table 1:*** Parameter mean values and their corresponding standard deviations in both subgroups with an average SpO_2_ <90 and SpO_2_ ≥ 90 used in calculating Mahalanobis. HR: heart rate, RR: respiratory rate, SpO_2_: oxygen saturation, rSO_2_: cerebral oxygen saturation, IBP: mean invasive blood pressure.

| **Parameter mean** | SpO2 < 90 | SpO_2_ ≥ 90 |
| --- | --- | --- |
| HR, beats/min, mean (standard dev.) | 157 (18.4) | *144 (18.6)* |
| RR, breaths/min, mean (standard dev.) | 34 (8.3) | 35 (8.7) |
| SpO_2_, % , mean (standard dev.) | 75 (7.7) | 96 (3.2) |
| rSO_2_, % , mean (standard dev.) | 56 (10.1) | 72 (12.0) |
| IBP, mm hg , mean(standard dev.) | 53 (8.0) | 54 (9.8) |

***Appendix 2, Table 2:*** Parameter correlation matrix in the subgroup with an average SpO_2_ ≥ 90. Correlations were calculated using the Spearman method on complete data. HR: heart rate, RR: respiratory rate, SpO_2_: oxygen saturation, rSO_2_: cerebral oxygen saturation, IBP: mean invasive blood pressure.

| **SpO_2_ ≥ 90** | HR | RR | SpO_2_ | rSO_2_ | IBP |
| --- | --- | --- | --- | --- | --- |
| HR  RR  SpO2  rSo2  IBP | -  0.12  -0.06  -0.21  -0.09 | 0.12  -  0.01  -0.02  0.00 | -0.06  0.01  -  0.17  0.08 | -0.21  -0.02  0.17  -  0.17 | -0.09  0.00  0.08  0.17  - |

***Appendix 2, Table 3:*** Parameter correlation matrix in the subgroup with an average SpO_2_ <90. Correlations were calculated using the Spearman method on complete data. HR: heart rate, RR: respiratory rate, SpO_2_: oxygen saturation, rSO_2_: brain oxygen saturation, IBP: mean invasive blood pressure.

| **SpO2 < 90** | HR | RR | SpO_2_ | rSO_2_ | IBP |
| --- | --- | --- | --- | --- | --- |
| HR  RR  SpO_2_  rSO_2_  IBP | -  0.04  -0.20  -0.11  -0.06 | 0.04  -  -0.10  -0.01  0.05 | -0.20  -0.10  -  0.40  0.14 | -0.11  -0.01  0.40  -  -0.08 | -0.06  0.05  0.14  -0.08  - |
