# Supplementary material for: Continuous Data-Driven Monitoring in Critical Congenital Heart Disease: Clinical Deterioration Model Development
Source: JMIR Cardio. 2023 May 16;7:e45190. doi: 10.2196/45190 (PMC10230358; doi:10.2196/45190)
Supplement: Multimedia Appendix 3 [file cardio_v7i1e45190_app3.docx]

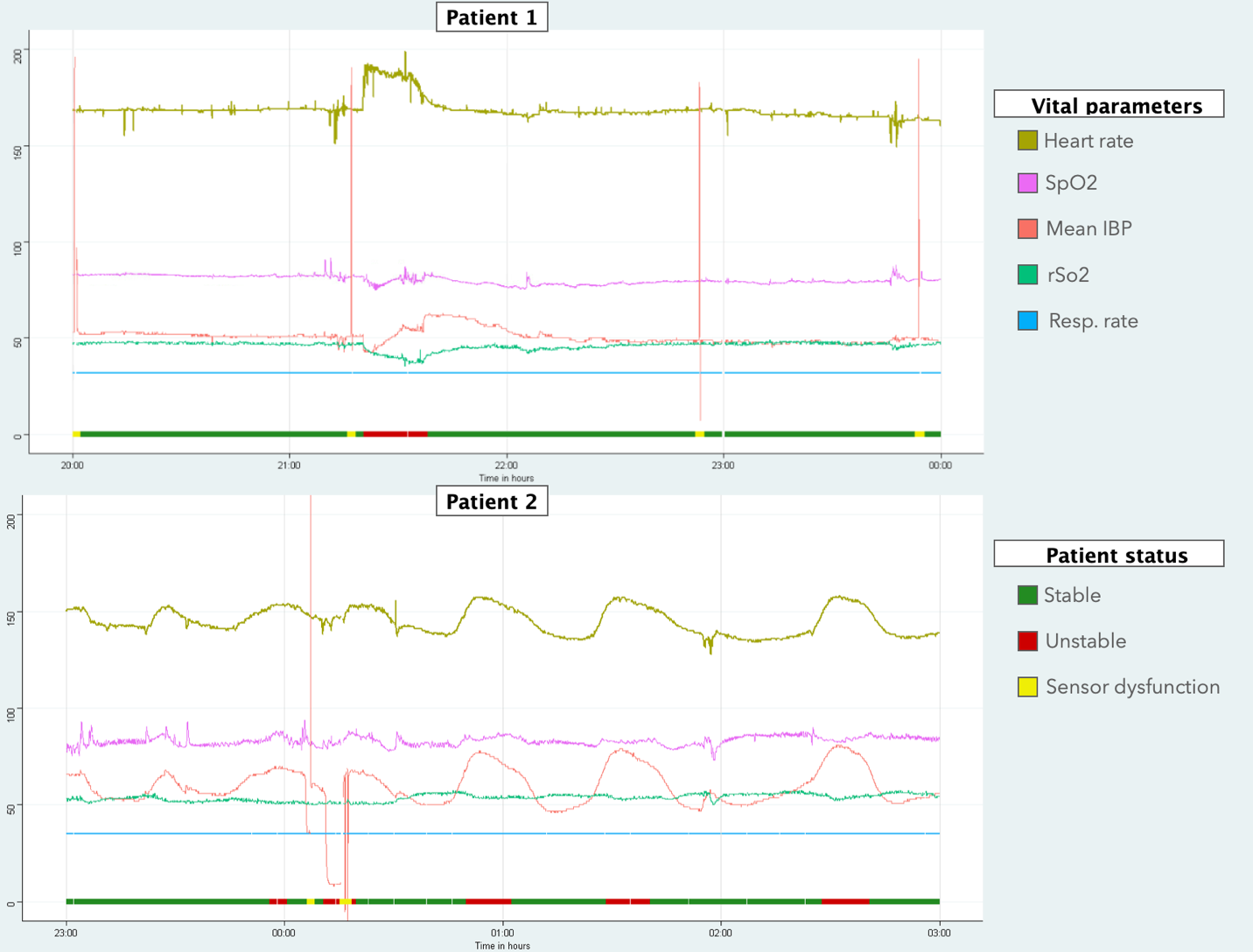
*Appendix 3, figure A: Clinical deterioration detection visualized in patients 1 and 2 during four hours of their admission. The lower bar in each graph depicts the current patient status as analyzed by the algorithm. IBP: mean invasive blood pressure, SpO_2_: oxygen saturation, rSO_2_: cerebral oxygen saturation.*

*Appendix 3, figure A: Clinical deterioration detection visualized in patients 3 and 4 during four hours of their admission. The lower bar in each graph depicts the current patient status as analyzed by the algorithm. IBP: mean invasive blood pressure, SpO_2_: oxygen saturation, rSO_2_:*
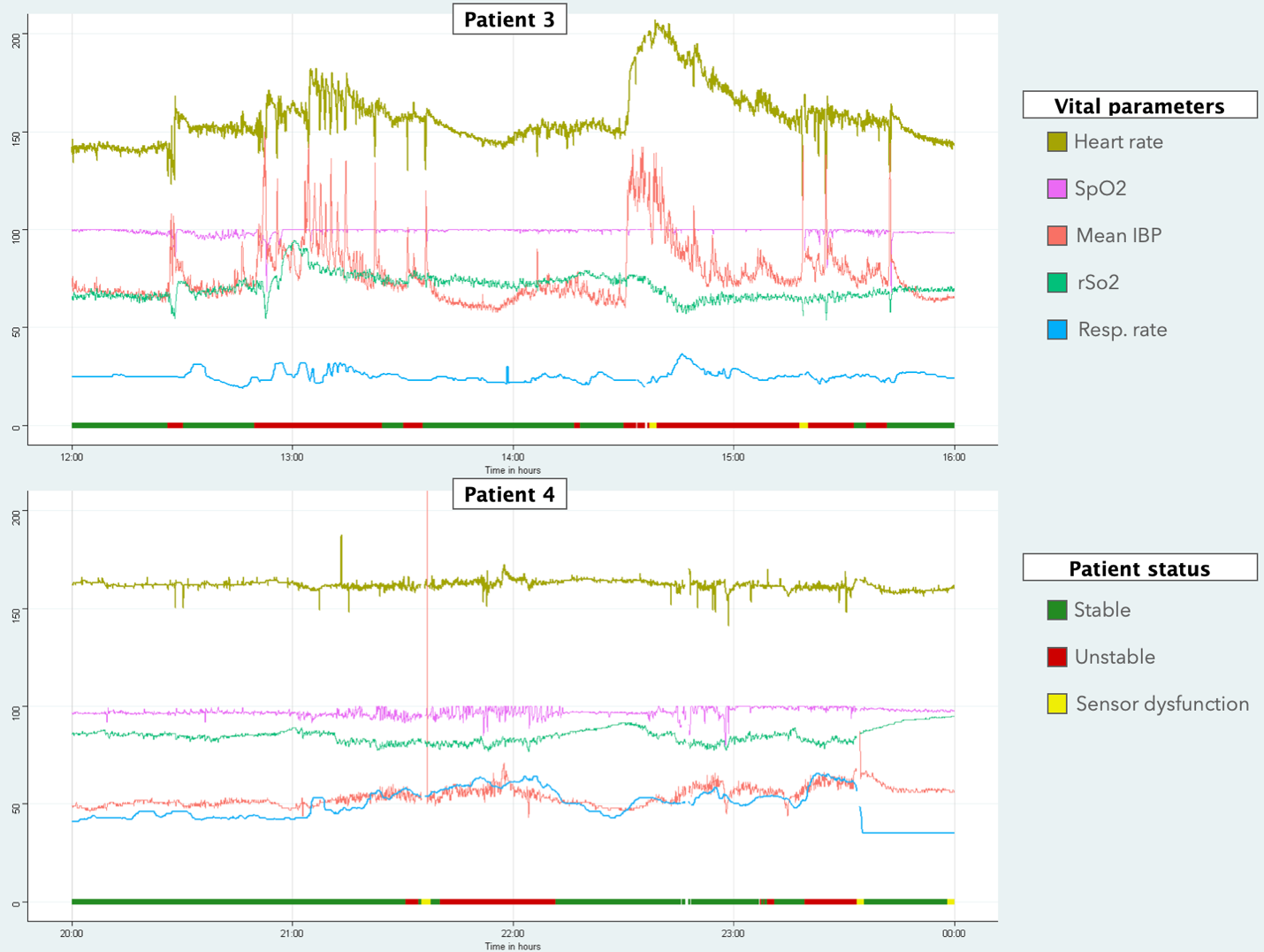
*cerebral oxygen saturation.*
