# Supplementary material for: Continuous Data-Driven Monitoring in Critical Congenital Heart Disease: Clinical Deterioration Model Development
Source: JMIR Cardio. 2023 May 16;7:e45190. doi: 10.2196/45190 (PMC10230358; doi:10.2196/45190)
Supplement: Multimedia Appendix 4 [file cardio_v7i1e45190_app4.docx]

| **Appendix 4, table 1:** Cardiac diagnosis and surgical procedure of included patients. *CHD: Congenital Heart Disease*. | | | |
| --- | --- | --- | --- |
| Characteristics | | | Patients (n, %)  Total = 78 |
| CHD Diagnosis | | |  |
|  | Aortic Arch Anomalies | | 22 (28) |
|  |  | Hypoplastic Aortic Arch | *9* |
|  |  | Coarctatio Aortae | *5* |
|  |  | Truncus Arteriosus | *4* |
|  |  | Interrupted Aortic Arch | *4* |
|  | Transposition of the great arteries | | 21 (27) |
|  | Hypoplastic Left Heart Syndrome | | 19 (24) |
|  | Hypoplastic Left Heart Complex | | 5 (6) |
|  | Complex atrial and/or ventricular septal defects | | 5 (6) |
|  | Tetralogie of Fallot | | 2 (3) |
|  | Other *(e.g. double inlet/outlet ventricles, m. Ebstein)* | | 4 (5) |
|  |  | |  |
| Surgical procedure | | |  |
|  | Norwood procedure | | 22 (28) |
|  | Arterial Switch procedure | | 20 (26) |
|  | Aortic arch reconstruction | | 17 (22) |
|  | Biventricular repair | | 8 (10) |
|  | Shunt placement | | 5 (6) |
|  | Damus-Kaye-Stansel procedure | | 3 (4) |
|  | Ross-Konno procedure | | 3 (4) |
|  |  | |  |
